# Multiple schwannomas synchronously occurring in the porta hepatis, liver, and gallbladder: first case report

**DOI:** 10.1097/MD.0000000000004378

**Published:** 2016-08-19

**Authors:** Shao-yan Xu, Hua Guo, Yan Shen, Ke Sun, Hai-yang Xie, Lin Zhou, Shu-sen Zheng, Wei-lin Wang

**Affiliations:** a Division of Hepatobiliary and Pancreatic Surgery, Department of Surgery; b Key Laboratory of Combined Multi-organ Transplantation, Ministry of Public Health; c Key Laboratory of Organ Transplantation; d Collaborative Innovation Center for Diagnosis and Treatment of Infectious Diseases; e Department of pathology (KS), First Affiliated Hospital, School of Medicine, Zhejiang University, Zhejiang Province, Hangzhou,China.

**Keywords:** case report, gallbladder, liver, porta hepatis, schwannomas

## Abstract

**Background:**

Schwannomas are mesenchymal neoplasms that arise from Schwann cells with low malignant potential. Schwannomas originating from the porta hepatis or intra-abdominal organs are extremely rare. To our knowledge, multiple schwannomas synchronously occurring in the porta hepatis, liver, and gallbladder have not been reported so far and we first report one in the present case.

**Case summary:**

A 31-year-old female was referred to our hospital because of repeated abdomen discomfort, slight abdominal distension, and occasional abdominal pain for seven years. Ultrasound and computed tomography and magnetic resonance cholangiopancreatography found multiple intrahepatic and extrahepatic cystic lesions with the dilation of intrahepatic and extrahepatic bile ducts. By exploratory laparotomy, multiple tumors were found in the porta hepatis, liver, and gallbladder, the biggest one was 11 × 6 cm in size. We completely resected these tumors combined with the left lateral liver lobe, gallbladder, and the invaded left and right hepatic arteries, and then severed vessels were reconstructed. Microscopically, the tumor cells were spindle shaped and palisading arrangement. Atypical cells or signs of malignancy were not found. Immunohistochemical investigation showed the protein S-100 was positive, while SMA, CD34, and CD117 negative. Finally, these tumors were diagnosed as schwannomas in the porta hepatis, liver, and gallbladder. The patient is followed-up for 70 months and has been doing well without any complications.

**Conclusion:**

We report the first patient with multiple schwannomas synchronously occurring in the porta hepatis, liver, and gallbladder. Accurate preoperative diagnosis of these tumors is difficult. Due to closely adhering to the surrounding important tissues, complete removal is challenging.

## 1 Introduction

Schwannomas are neoplasms that originate from Schwann cells of the peripheral nerves.^[1]^ Patients between 20 and 50 years old were most reported with equal frequency in male and female.^[2]^ More than 90% Schwannomas are benign and manifest about 5% of benign soft-tissue neoplasm.^[2]^ Due to the lack of specific clinical and imaging characteristics, it is challenging to make an accurate preoperative diagnosis. Although schwannomas can develop in any part of the body, the most common sites are the head, neck, and flexor surfaces of the extremities.^[3]^ The tumors generally have a single place of origin and only 10% originate from multiple locations.^[4]^ Schwannomas occurring in the porta hepatis or abdominal organs are rare. To our knowledge, only 18, 15, and 6 cases of schwannomas have been reported in the liver,^[5,6]^ porta hepatis,^[7]^ and gallbladder,^[8]^ respectively. However, multiple schwannomas synchronously occurring in the porta hepatis and abdominal organs have not been reported so far. In the present case, we report the first one in a 31-year-old female. Surgery may be the optimal treatment for schwannomas and patients with benign schwannomas generally have a good prognosis. The patient of the present study underwent a successful surgery and recovered well.

## 2 Case report

**Figure 1:**
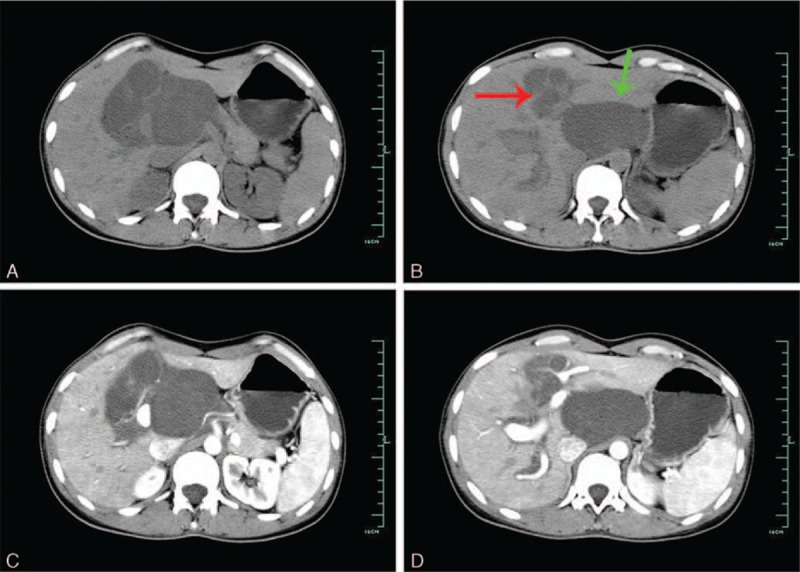
An unenhanced CT imaging showed multiple cystic lesions in the left liver (red arrow), as well as the space between left kidney and body of the stomach (green arrow) (A and B). On the contrast-enhanced CT, these lesions were not obviously enhanced (C and D). CT = computed tomography.

**Figure 2:**
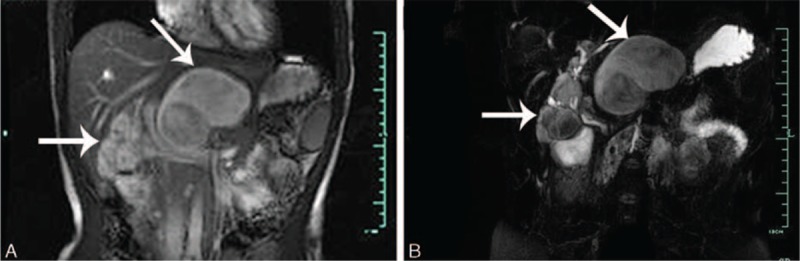
MRCP showed multiple inhomogeneous hyperintense lesions on T2 weighted images in the hepatic portal and left liver (arrow) with the dilation of intrahepatic and extrahepatic bile ducts. MRCP = magnetic resonance cholangiopancreatography.

On June 15 2010, a 31-year-old female was referred to our hospital because of repeated abdomen discomfort, slight abdominal distension, and occasional abdominal pain for 7 years. The physical examination evidenced a subxiphoid mass with mild tenderness, 10 cm in diameter. There was no history of weight loss, trauma, nor family history of cancer. Laboratory results: Total bilirubin was 25.80 μmol/L (0–21), direct bilirubin 10 μmol/L (0–5), and indirect bilirubin 15.8 μmol/L (3–14). Other abnormal laboratory results were not found. Ultrasound (US) showed multiple intrahepatic and extrahepatic cystic lesions with intrahepatic bile ducts dilatated. An unenhanced computed tomography (CT) imaging showed multiple cystic lesions in the left liver as well as the space between left kidney and body of the stomach (Fig. 1A and B). On the contrast-enhanced CT, these lesions were not obviously enhanced (Fig. 1C and D). Magnetic resonance cholangiopancreatography showed multiple inhomogeneous hyperintense lesions on T2 weighted images in the hepatic portal and left liver with the dilation of intrahepatic and extrahepatic bile ducts (Fig. 2). However, an accurate diagnosis could not be obtained and these lesions were primarily considered benign occupations in the liver and abdominal cavity.

After sufficient preoperation preparation, an exploratory laparotomy was performed. A 10 × 15 cm mass was found in the porta hepatis, wrapping the whole hepatoduodenal ligament, invading the common bile duct, left and right hepatic bile ducts as well as the right and left hepatic arteries. There was also a mass located in the left lateral liver lobe, 6 cm in diameter, invading the hepatic portal. Another mass grew from the gallbladder wall and invaded the right hepatic bile duct, 5 cm in diameter. The mass in porta hepatis was lobulated and connected with the masses in liver and gallbladder via tubular tissues. We carefully isolated and completely resected these masses combined with the gallbladder and invaded left lateral liver lobe. However, the left and right hepatic arteries were also invaded by tumors and hard to be separated. We isolated, ligated, and resected the invaded left and right hepatic artery and reconstructed the severed vessels. Intraoperative frozen pathology showed soft tissue tumors and liposarcomas were first considered. After operation, the patient recovered smoothly and left the hospital 9 days later.

**Figure 3:**
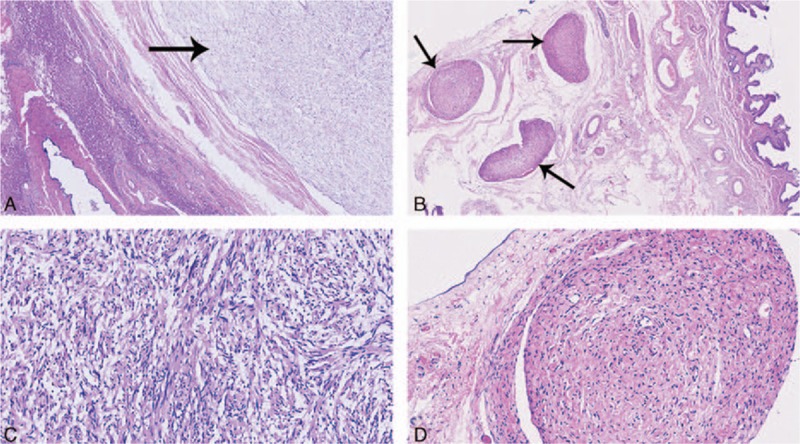
Microscopically, multiple lesions were found in the liver (arrow) (A) and gallbladder (arrow) (B) with thin capsules (×40). The tumor cells were spindle shaped and palisading arrangement. Both hypercellular and hypocellular areas were visible, while atypical cells or signs of malignancy were not found (C, D) (×200).

**Figure 4:**
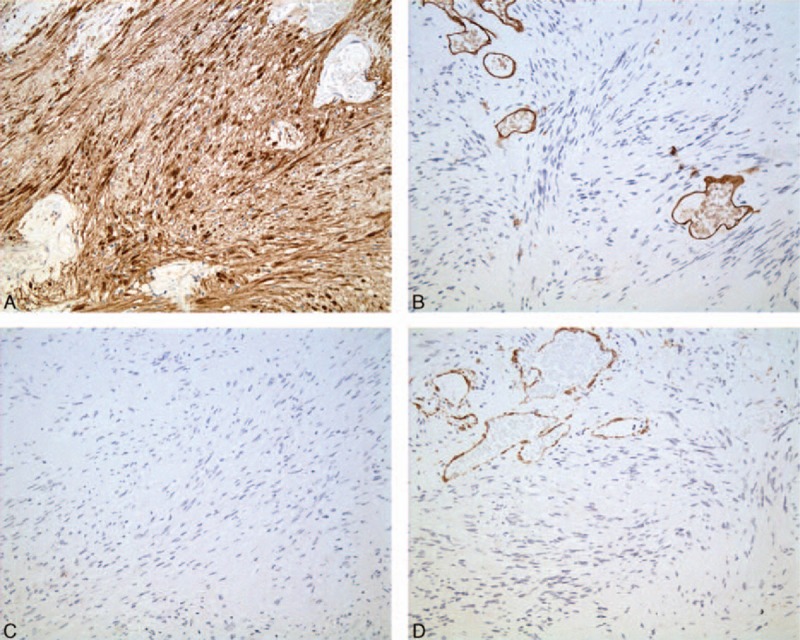
Immunohistochemical stains showed the protein S-100 was positive (A), while CD34 (B), CD117 (C), and SMA (D) negative (×200).

Macroscopically, one big tumor was located in the porta hepatis sized 11 × 6 cm. Multiple tumors with different sizes were also found in the liver and gallbladder. The biggest one in the liver was 9 × 7.5 cm and the biggest one in the gallbladder 5 × 3.5 cm. These tumors were canary yellow. Microscopically, multiple lesions with thin capsules were found in the liver (Fig. 3A) and gallbladder (Fig. 3B). The tumor cells were spindle shaped and palisading arrangement. Both hypercellular and hypocellular areas were visible, while atypical cells or signs of malignancy were not found (Fig. 3C and 3D). Immunohistochemical stains showed the protein S-100 was positive (Fig. 4A), while CD34, CD117, and SMA negative (Fig. 4B–4D). Finally, these tumors were diagnosed as multiple schwannomas synchronously in the porta hepatis, liver, and gallbladder. The patient is followed-up for 70 months and has been doing well without any complications.

## 3 Discussion

Schwannomas originate from the nerve sheaths of the peripheral nerves, which can occur in different ethnic groups worldwide with equal frequency in male and female and were most reported in patients between 20 and 50 years old.^[2]^ The exact etiology and pathogenesis of schwannomas remain unclear. Secondary degenerations including cyst formation, hemorrhage, calcification, and hyalinization can sometimes be shown.^[9]^ The most common sites of schwannomas are head, neck and flexure surfaces of the extremities.^[10]^ In the abdominal cavity, schwannomas are most reported in the retroperitoneum^[11]^ and gastrointestinal tract.^[12,13]^ The occurrence of schwannomas in the porta hepatis,^[7]^ gallbladder,^[8]^ pancreas,^[14]^ or liver^[15]^ is extremely rare. To our knowledge, only 18, 15, and 6 cases of schwannomas involving the liver,^[5,6]^ porta hepatis, and gallbladder,^[8]^ respectively, have been reported. However, multiple benign schwannomas synchronously occurring in the porta hepatis, liver, and gallbladder have not been reported so far and we report the first case.

Du to lacking of specific clinical and imaging features, accurate preoperative diagnosis of the tumor is a tremendous challenge. Histopathological and immunohistochemical examinations are essential for definitive diagnosis. Microscopically, a typical schwannoma is composed of Antoni A and Antoni B areas. The Antoni A area is hypercellular and characterized by closely packed spindle cells with occasional nuclear palisading and Verocay bodies. The Antoni B area is hypocellular and occupied by loosely arranged tumor cells with abundant myxoid stroma.^[16]^ Immunohistochemically, schwannomas are strongly positive for S-100.^[17]^ However, this group of proteins are not totally specific for this tumor. S-100 proteins may be present in melanocytes, chondrocytes, adipocytes, myoepithelial cells, macrophages, Langerhans cells, dendritic cells, and some breast epithelial cells.^[18]^ Consequently, S-100 can be found in melanomas, neurofibromas, paraganglioma stromal cells, histiocytoma, and clear cell sarcomas.^[19]^ So, the diagnosis of schwannoma should be based on the results of both immunohistochemical and histopathological examinations.

Multiple imaging modalities including US, CT, magnetic resonance imaging could be used to determine the lesion limits and establish a probable diagnosis. However, the definitive preoperative diagnosis is hard to achieve due to the lack of specific imaging features. On US, schwannomas are usually hypodense masses with well-defined boundary, and no intralesional blood flow signals were visible on Color Doppler US.^[6]^ On unenhanced CT scan, schwannomas are generally hypoechoic and well defined with fibrous capsules. Schwannomas with high Antoni A areas appear inhomogeneous. Antoni B areas of schwannomas show low density and appear cystic with multiseptum due to low cellularity.^[8]^ On contrast-enhanced CT, Antoni A areas are well-enhanced due to the increased vascularity, while Antoni B areas are unenhanced due to less vascularity.^[8]^ On magnetic resonance imaging, schwannomas appear hypointense on T1 weighted images and inhomogeneous hyperintense on T2 weighted images.^[20]^ Although these imaging findings are not typical characteristics, they are helpful in treatment planning because of providing information on the invasion of other structures. Endoscopic ultrasound-guided fine needle aspiration may greatly contribute to precise preoperative diagnosis and provide useful information to guide therapy. In one report, three cases of asymptomatic retroperitoneal lesions were accurately diagnosed as benign schwannomas by preoperative endoscopic ultrasound-guided fine needle aspiration and avoided surgical resection.^[21]^

Surgery may be curative by completely removing the tumor. In the present case, by laparotomy, we found multiple lesions synchronously occurring in the porta hepatis, liver, and gallbladder. These lesions intensively adhered to the surrounding tissues and even invaded the hepatic arteries, common bile duct, and left and right hepatic bile ducts. We completely resected these tumors combined with the left lateral liver lobe, gallbladder, and the invaded left and right hepatic arteries. Immediately, the reconstruction of severed vessels was performed. Histopathological and immunohistochemical examinations of surgical specimens showed multiple schwannomas synchronously occurring in the porta hepatis, liver, and gallbladder.

## 4 Conclusion

This is the first case of multiple benign schwannomas synchronously occurring in the porta hepatis, liver, and gallbladder. It is a huge challenge to definitively determine intra-abdominal schwannomas preoperatively, despite the utilization of multiple imaging modalities. Definitive diagnosis of schwannomas originating from intra-abdominal tissues or organs nearly can only be obtained by histopathological and immunohistochemical examinations. Early detection of the tumor is important and complete excision is the optimal treatment. In the present case, due to closely adhering to the surrounding important tissues, complete removal is challenging. We resected these schwannomas successfully and the patient has a good prognosis.

## References

[1] Das Gupta TK Brasfield RD Strong EW Benign solitary Schwannomas (neurilemomas). *Cancer* 1969; 24:355–366. 5796779 10.1002/1097-0142(196908)24:2<355::aid-cncr2820240218>3.0.co;2-2

[2] Das Gupta TK Brasfield RD Tumors of peripheral nerve origin: benign and malignant solitary schwannomas. *CA Cancer J Clin* 1970; 20:228–233. 4316984 10.3322/canjclin.20.4.228

[3] Ariel IM Tumors of the peripheral nervous system. *CA Cancer J Clin* 1983; 33:282–299. 6413007 10.3322/canjclin.33.5.282

[4] Fenoglio L Severini S Cena P Common bile duct schwannoma: a case report and review of literature. *World J Gastroenterol* 2007; 13:1275–1278. 17451214 10.3748/wjg.v13.i8.1275 PMC4147008

[5] Hayashi M Takeshita A Yamamoto K Primary hepatic benign schwannoma. *World J Gastrointest Surg* 2012; 4:73–78. 22530081 10.4240/wjgs.v4.i3.73 PMC3332224

[6] Ota Y Aso K Watanabe K Hepatic schwannoma: imaging findings on CT, MRI and contrast-enhanced ultrasonography. *World J Gastroenterol* 2012; 18:4967–4972. 23002371 10.3748/wjg.v18.i35.4967 PMC3447281

[7] Yin SY Zhai ZL Ren KW Porta hepatic schwannoma: case report and a 30-year review of the literature yielding 15 cases. *World J Surg Oncol* 2016; 14:103. 27038921 10.1186/s12957-016-0858-9 PMC4818894

[8] Liu LN Xu HX Zheng SG Solitary schwannoma of the gallbladder: a case report and literature review. *World J Gastroenterol* 2014; 20:6685–6690. 24914396 10.3748/wjg.v20.i21.6685 PMC4047360

[9] Honjo Y Kobayashi Y Nakamura T Extrahepatic biliary schwannoma. *Dig Dis Sci* 2003; 48:2221–2226. 14705833 10.1023/b:ddas.0000004531.97727.66

[10] Le Guellec S [Nerve sheath tumours]. *Ann Pathol* 2015; 35:54–70. 25541115 10.1016/j.annpat.2014.11.008

[11] Ratnagiri R Mallikarjun S Retroperitoneal ancient schwannoma: two cases and review of literature. *J Cancer Res Ther* 2014; 10:368–370. 25022395 10.4103/0973-1482.136660

[12] Prevot S Bienvenu L Vaillant JC Benign schwannoma of the digestive tract: a clinicopathologic and immunohistochemical study of five cases, including a case of esophageal tumor. *Am J Surg Pathol* 1999; 23:431–436. 10199472 10.1097/00000478-199904000-00007

[13] Tao K Chang W Zhao E Clinicopathologic features of gastric schwannoma: 8-year experience at a single institution in China. *Medicine (Baltimore)* 2015; 94:e1970. 26559271 10.1097/MD.0000000000001970 PMC4912265

[14] Nishikawa T Shimura K Tsuyuguchi T Contrast-enhanced harmonic EUS of pancreatic schwannoma. *Gastrointest Endosc* 2016; 83:463–464. 26341855 10.1016/j.gie.2015.08.041

[15] Ozkan EE Guldur ME Uzunkoy A A case report of benign schwannoma of the liver. *Intern Med* 2010; 49:1533–1536. 20686285 10.2169/internalmedicine.49.3486

[16] Lee WH Kim TH You SS Benign schwannoma of the liver: a case report. *J Korean Med Sci* 2008; 23:727–730. 18756066 10.3346/jkms.2008.23.4.727 PMC2526407

[17] Weiss SW Langloss JM Enzinger FM Value of S-100 protein in the diagnosis of soft tissue tumors with particular reference to benign and malignant Schwann cell tumors. *Lab Invest* 1983; 49:299–308. 6310227

[18] Cochran AJ Wen DR S-100 protein as a marker for melanocytic and other tumours. *Pathology* 1985; 17:340–345. 2995906 10.3109/00313028509063777

[19] Turusov VS [Protein S-100 in the histological diagnosis of tumors]. *Arkh Patol* 1990; 52:71–78. 2186722

[20] Momtahen AJ Akduman EI Balci NC Liver schwannoma: findings on MRI. *Magn Reson Imaging* 2008; 26:1442–1445. 18499377 10.1016/j.mri.2008.04.001

[21] Kudo T Kawakami H Kuwatani M Three cases of retroperitoneal schwannoma diagnosed by EUS-FNA. *World J Gastroenterol* 2011; 17:3459–3464. 21876639 10.3748/wjg.v17.i29.3459 PMC3160573

